# Peripheral immune reactions following human traumatic spinal cord injury: the interplay of immune activation and suppression

**DOI:** 10.3389/fimmu.2024.1495801

**Published:** 2024-11-27

**Authors:** Hanne Coenen, Veerle Somers, Judith Fraussen

**Affiliations:** Department of Immunology and Infection, Biomedical Research Institute, UHasselt – Hasselt University, Hasselt, Belgium

**Keywords:** spinal cord injury, peripheral immune reaction, inflammation, autoimmunity, immunosuppression

## Abstract

Traumatic spinal cord injury (SCI) damages the nerve tissue of the spinal cord, resulting in loss of motor and/or sensory functions at and below the injury level. SCI provokes a long-lasting immune response that extends beyond the spinal cord and induces changes in the composition and function of the peripheral immune system. Seemingly contradictory findings have been observed, as both systemic immune activation, including inflammation and autoimmunity, and immune suppression have been reported. Differences in the levels and functions of various cell types and components of both the innate and adaptive immune system supporting these changes have been described at (sub)acute and chronic stages post-injury. Further research is needed for a more comprehensive understanding of the peripheral immune reactions following SCI, their possible correlations with clinical characteristics, and how these immune responses could be targeted to facilitate the therapeutic management of SCI. In this review, we provide an overview of the current literature discussing changes in the peripheral immune system and their occurrence over time following a traumatic SCI.

## 1 Introduction

Spinal cord injury (SCI) is defined as damage to the nerve tissue of the spinal cord, resulting in reduction or loss of sensory and/or motor function (1). Globally, more than 15 million people are suffering from SCI, of which up to 90% of cases are of traumatic origin (2, 3). In traumatic SCI, the primary injury is caused by an external physical force affecting the spinal cord, such as a fall, motor vehicle accident, sports-related accident, or violence (4–6). This trauma results in acute cell damage and cell death in the surrounding neuronal and vascular tissues. Subsequently, a secondary injury cascade is initiated, characterized by inflammatory cell infiltration, ischemia, edema, hemorrhage, and the release of cytotoxic products, resulting in further spinal cord damage and neurological dysfunction (1, 7). In addition to the pathophysiological subdivision of traumatic SCI into primary and secondary injuries, it can also be divided into temporal phases. Although different classifications are used in literature, generally, acute and subacute phases last hours to weeks, whereas the chronic phase refers to six months post-injury and beyond (1, 8).

During the secondary injury phase, the destruction of the blood-spinal cord barrier following the initial trauma allows the recruitment of various immune cells into the injured spinal cord. Local inflammation of the spinal cord in the early stages following SCI is mediated by the innate immune system. As demonstrated in human post-mortem SCI spinal cords, neutrophils are the first immune cells to reach the areas of injury, peaking in number at 1-3 days and remaining increased up to 10 days post-injury (9, 10). Subsequently, resident microglia are activated and monocytes/macrophages infiltrate the spinal cord. At 5-10 days post-injury and beyond, microglia and macrophages are the predominant inflammatory cells in the spinal cord (9–11). Since it takes longer for the adaptive immune system to initiate an immune response via processes of antigen recognition, activation, proliferation, and clonal expansion, few lymphocytes populate the lesion site in the acute stages following injury (11). Nevertheless, at one week to months post-injury, CD8^+^ and CD4^+^ T cells could be detected in human post-mortem spinal cord lesions (9, 10). In addition, for a subset of SCI patients in the subacute phase, a pronounced infiltration of B cells and active plasma cells was observed in human autopsy spinal cord tissues at 15 days up to 36 days post-injury (10, 12).

Changes in the immune system are not limited to the accumulation and activation of immune cells at the site of injury. Instead, SCI also affects peripheral immune function and composition, as demonstrated in both SCI animal models and SCI patients. Here, we focus on human studies since species-specific differences have been reported in immune responses following SCI (13–15), contributing to a lack of clinical translation of the findings in experimental SCI models. Contradictory findings have been reported in SCI patients at various stages following the injury with, on the one hand, inflammation and autoreactivity, but on the other hand, immunosuppression. In this review, we discuss the current literature supporting both perspectives regarding the peripheral immune system in traumatic SCI patients. An overview of the studies that are covered in this review is given in **Supplementary Tables 1**, **2**.

## 2 Activation of the immune system

**Figure 1:**
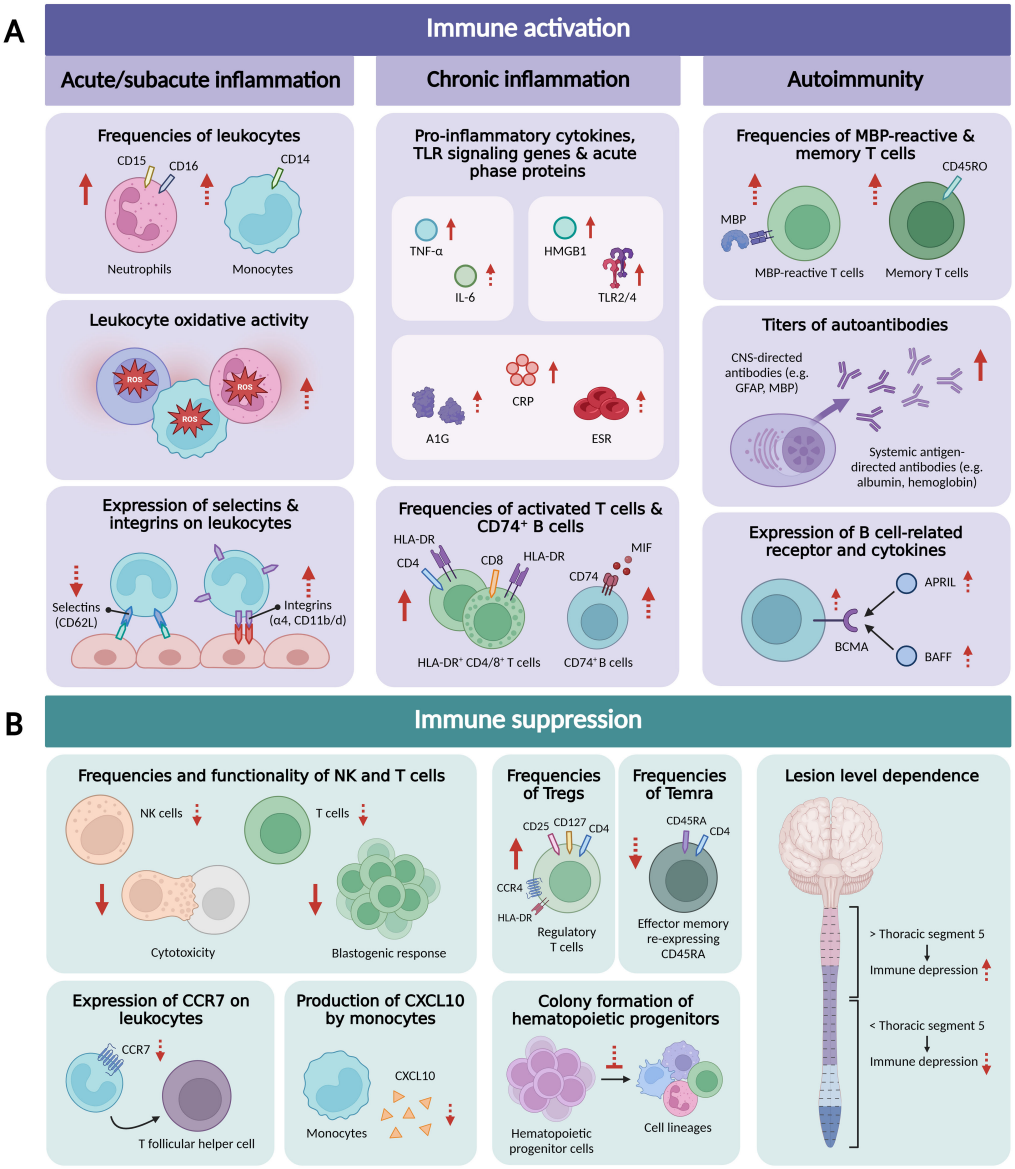
Overview of research findings related to peripheral immune activation **(A)**, including acute/subacute systemic inflammation, chronic systemic inflammation, and autoimmunity, and peripheral immune suppression **(B)** in traumatic SCI patients. Arrows in dashed line indicate that only one study demonstrated this finding or that contrasting findings were reported. *APRIL, a proliferation-inducing ligand; A1G, alpha-1 globulin; BAFF, B-cell activating factor; BCMA, B-cell maturation antigen; CCR7, C-C chemokine receptor type 7; CNS, central nervous system; CRP, C-reactive protein; CXCL10, C-X-C motif chemokine ligand 10; ESR, erythrocyte sedimentation rate; GFAP, glial fibrillar acidic protein; HLA-DR, Human Leukocyte Antigen DR isotype; HMGB1, High Mobility Group Box 1 protein; IL-6, interleukin 6; MBP, myelin basic protein; MIF, macrophage migration inhibitory factor; NK, natural killer; ROS, reactive oxygen species; Temra, CD4^+^ effector memory T cells re-expressing CD45RA; TLR, Toll-like receptor; TNF-α, tumor necrosis factor-α.* Figure created with BioRender.com.

Emerging experimental and clinical data indicate that SCI triggers activation of the peripheral immune system, with signs of systemic inflammation and autoreactivity, at various stages following the initial trauma (**Figure 1A**).

### 2.1 Acute and subacute systemic inflammation

Several studies have demonstrated an acute systemic increase in white blood cell numbers following human SCI. Total leukocyte counts greater than reference values (17.1 ± 1.4×10^9^ vs. 4–10×10^9^ cells/L) and significantly higher compared to trauma controls (TC), defined as patients with a trauma not involving central nervous system (CNS) injury, were observed at 3.5 ± 1 hours (h) and one week after injury, respectively (16, 17). This (sub)acute increase was mainly attributed to neutrophilia, as neutrophil counts were also strongly elevated above clinical reference ranges at 3.5 ± 1 h post-injury (14.8 ± 1.3×10^9^ vs. 2–7.5×10^9^ cells/L) (16). Another study also reported a transient but significant increase in neutrophil counts compared to reference values within the first 24 h following SCI in both analyzed cohorts, i.e. an exploration and an independent validation patient cohort (18). Circulating monocyte numbers were only significantly elevated in the exploration cohort on the first day and one week post-injury, and not in the validation cohort (18). This discrepancy could be due to differences in the sizes of the exploration cohort (n = 161) and validation cohort (n = 49). In addition, monocyte numbers were reported to be at the upper reference limit at 3.5 ± 1 h in SCI patients (0.8 ± 0.1×10^9^ vs. 0.2–0.8×10^9^ cells/L) (16). When analyzing monocyte subsets, those subsets with the highest phagocytosis capacity, namely classical (CD14^+^CD16^-^) and intermediate (CD14^+^CD16^+^) monocyte populations, were significantly increased in blood samples collected 0-3 days post-injury compared to a healthy control (HC) group, whereas no differences were observed for non-classical monocytes (CD14^-/lo^CD16^+^) (19, 20).

Leukocytosis (increased blood leukocyte levels) in response to physical trauma is associated with the activation of leukocytes in the circulation, increasing their oxidative and phagocytic-like activity and migration capacity (21, 22). Such activation not only primes the cells to exacerbate the primary injury upon entry into the spinal cord, but the leukocytes can also infiltrate and damage organs and tissues that were initially unaffected, such as the lungs and kidneys (23–25). Circulating neutrophils and monocytes demonstrated increased cellular oxidation at 12 h, 24 h, and one week following injury compared to HC and TC (16). Moreover, free radical production and the activity of myeloperoxidase (MPO), an enzyme that generates highly reactive products with antimicrobial actions, were measured in leukocyte homogenates. Both were significantly increased in SCI patients compared to TC within the first two weeks following injury (16). Lastly, the protein expression of oxidative enzymes, nicotinamide adenine dinucleotide phosphate (NADPH) oxidase subunit gp91^phox^ and inducible nitric oxide synthetase (iNOS), was 20–25% higher in leukocyte homogenates and leukocytes of blood smears of SCI patients than in those of TC (16). This further confirms elevated leukocyte oxidative activity.

The potential of leukocytes to infiltrate and damage organs and tissues is related to their expression of various selectins and integrins. Hereby, selectins initiate the transient attachment and rolling of leukocytes along the endothelial surface. Subsequently, integrins are responsible for the firm adhesion of the cells to the endothelium, allowing their migration into neighboring tissue (26). Changes in the surface expression of these adhesion molecules on human peripheral leukocytes have been demonstrated after SCI. Although the percentage of human peripheral neutrophils and monocytes expressing L-selectins (CD62L) did not change in the 6 h to two-week period following SCI, the level of CD62L surface expression decreased markedly in SCI patients compared to HC (27). The authors hypothesized that this observation could be attributed to the shedding of CD62L following ligation to the endothelium, a process that has been shown to play an important role in regulating leukocyte rolling (28, 29). However, further research is needed to confirm this theory. Additionally, the expression of integrin subunits α4, CD11d, and CD11b on neutrophils and/or monocytes was significantly increased in SCI patients compared to HC and/or TC between 12 h and 2 weeks post-injury (27).

In addition to neutrophils and monocytes, another subset of innate immune cells, natural killer (NK) cells, has been related to acute SCI-induced inflammation. NK cells play an important regulatory role by secreting cytokines and have a high cytotoxic potential, being crucial to combat viral and bacterial infections (26). In blood samples collected within 24 h post-SCI, total NK cells were demonstrated to be present in higher frequencies and express higher levels of activation molecules (e.g. CD69 and human leukocyte antigen (HLA-DR)) compared to those of HC (30). As the percentage of highly cytokine producing CD56^bright^ NK cells was significantly lower in SCI patients compared to HC, the authors concluded that the NK cell population in SCI patients was mainly made up of cytotoxic CD56^dim^ cells (30). Nevertheless, this has been the only study to date that reported an increase in NK cells post-SCI (see section 3).

Altogether, these reports underscore the hypothesis that in the (sub)acute phase of SCI, circulating innate immune cell subsets increase in number and functional capacities, contributing to a systemic inflammatory response. This could lead to the accumulation of activated immune cells and organ damage, negatively impacting recovery from traumatic SCI.

### 2.2 Chronic systemic inflammation

In addition to an acute systemic inflammatory response, several studies also provided serologic evidence of a chronic level of immunoactivity in SCI patients.

Although blood levels of leukocytes were not elevated in chronic SCI patients compared to HC in three individual studies, increased levels of circulating pro-inflammatory cytokines have been reported (31–33). In a study in which the majority of included patients were in the chronic phase post-SCI, serum concentrations of the pro-inflammatory cytokines interleukin (IL)-6 and tumor necrosis factor (TNF)-α were significantly increased when compared to HC (34). A similar elevation of serum TNF-α was observed in a study population consisting solely of chronic SCI patients (35). For IL-6, other studies could not confirm elevated levels in the plasma/serum of chronic SCI patients compared to HC, although a non-significant increase was indicated in one study (36, 37). Interestingly, a systemic enrichment of genes related to Toll-like receptor (TLR) signaling was observed in whole blood of both acute and chronic SCI patients using RNA sequencing (19, 38). TLRs are pattern-recognition receptors expressed in immune cells that recognize conserved pathogen-associated molecular patterns (PAMPs) during innate immune responses. This induces the transcription of pro-inflammatory cytokines, such as IL-6 and TNF-α, via activation of the nuclear factor kappa-light-chain-enhancer of activated B cells (NF-kB) signaling pathway (39). In SCI, High Mobility Group Box 1 protein (HMGB1), a pro-inflammatory alarmin, and its cell surface receptors, including TLR2 and TLR4, were significantly elevated in whole blood at both acute (0-3 days post-injury) and chronic (six months post-injury) time points compared to HC (19). Additionally, significantly elevated levels of HMGB1 were demonstrated in plasma and whole blood samples of acute (≤1 week post-injury) and chronic (≥1 year post-injury) SCI patients compared to HC (38, 40).

Circulating levels of acute phase reactants, a class of proteins whose blood plasma concentration indicates the presence of an active inflammatory process (41), have also been analyzed in chronic SCI patients. Mean C-reactive protein (CRP) levels were significantly higher in chronic SCI patients than in HC (37). This increase in CRP levels has been confirmed by other research groups (42–44). Moreover, increased levels of alpha-1 globulin (A1G) and erythrocyte sedimentation rate (ESR) have also been reported in chronic SCI patients (43). Interestingly, pro-inflammatory cytokines, such as IL-6 and TNF-α, stimulate the acute phase response, which could be a possible link between the reported increase in these cytokines and acute phase proteins (39).

Another indication of chronic systemic inflammation is the increased activation of T cells observed in chronic SCI patients. Hereby, one study demonstrated elevated proportions of HLA-DR^+^ CD4^+^ and CD8^+^ T cells at 3 to 12 months post-injury compared to HC, although no significant differences in CD4^+^ and CD8^+^ T cell frequencies were shown between 0-3 days and 12 months, except for a significant reduction in CD4^+^ T cells at 3 months post-SCI (19). In lymphocytes, HLA-DR is a late-phase activation marker that is upregulated 24-48 h after cell activation (45). In the same study, significant upregulation of genes linked to T cell activation was observed up to 6 months following SCI compared to HC, which may be at the forefront of the increased levels of activated T cells in later stages (19). Similarly, in another study, chronic SCI patients (≥1 year post-injury) presented with significantly decreased frequencies of total T cells and CD4^+^ T cells, although frequencies of activated (HLA-DR^+^) CD4^+^ T cells were significantly elevated compared to HC (46). In addition to T cells, we demonstrated inflammation-related alterations in the B cell compartment in the chronic stage following SCI. Although no significant changes in total B cells have been reported in several studies (19, 32, 33, 47), we observed increased frequencies of CD74-expressing B cells in the peripheral blood of SCI patients compared to HC (47). Moreover, there was a trend towards increased CD74 expression on total B cells, as well as significantly increased CD74 expression on B cell subsets of subacute/chronic (>1 month post-injury) SCI patients compared with HC (47). CD74 functions as a receptor for the pro-inflammatory cytokine macrophage migration inhibitory factor (MIF), whose plasma levels are also increased in SCI patients at acute, subacute, and chronic stages post-injury (48–50).

The upregulation of TLR signaling, the elevated levels of pro-inflammatory cytokines and acute phase reactants, and the increased expression of activated T cells and CD74^+^ B cells suggest that SCI triggers chronic systemic inflammation.

### 2.3 Autoimmunity

Although SCI is not typically classified as an autoimmune disease, recent research in humans indicates that the disease can elicit autoimmune responses. Mainly adaptive immune reactions have been linked to this phenomenon.

It is speculated that similar to what has been described in multiple sclerosis (MS), autoreactive T cells can be activated upon encounter with myelin and other neurological breakdown products presented on antigen presenting cells, accumulate at the site of injury, and contribute to secondary inflammation (51). A first study demonstrated a higher precursor frequency of peripheral blood T cells reactive to myelin basic protein (MBP) in chronic SCI patients in comparison to HC, although this was not statistically significant (52). Still, MBP-reactive T cells of SCI patients showed several similarities with those of MS patients. MBP-reactive T cells of SCI and MS patients demonstrated similar precursor frequencies in peripheral blood mononuclear cells (PBMC), similar frequencies of cells reacting to the immunodominant 83-99 region of MBP, and a comparable pro-inflammatory cytokine profile with production of TNF-α and interferon (IFN)-γ (52). In addition, another research group indicated that *in vitro* MBP stimulated lymphocytes from the peripheral blood of chronic SCI patients showed a significantly higher proliferative response than those of HC (53). These studies point towards the presence and activation of myelin-reactive T cells in the circulation following a traumatic SCI. Their resemblances with MBP-reactive T cells of MS patients may point towards their potential involvement in spinal cord inflammation in SCI. However, their presence within the injured human spinal cord still needs to be demonstrated.

Due to their long lifespan and fast and efficient responses to antigens, memory T cells are of particular interest in the setting of autoimmune disorders (54). Interestingly, we showed that the distribution of CD4^+^ T cells shifted from more naive T cells (CD45RA^+^CD45RO^-^) in HC to more memory T cells (CD45RA^-^CD45RO^+^) in (sub)acute SCI patients (47). Furthermore, SCI patients showed trends towards increased frequencies of central memory (CD45RA^-^CCR7^+^) and effector memory (CD45RA^-^CCR7^-^) T cells, two main subpopulations of memory T cells (47). Elevated proportions of memory CD4^+^ T cell subsets have also been reported in autoimmune diseases such as MS and psoriasis, suggesting their role as critical mediators of autoimmunity (54, 55).

Evidence is also available for the involvement of B cells in SCI-induced autoimmunity. Elevated titers of autoantibodies, mostly directed against CNS proteins, have been reported in the peripheral blood of SCI patients. Increased levels of immunoglobulin (Ig)M antibodies against the CNS protein monosialotetrahexosylganglioside (GM1) have been reported in chronic SCI (>1 year post-injury) (35), while increased anti-GM1 IgG antibodies were described in both subacute and chronic SCI (34). Moreover, circulating levels of antibodies targeting glial fibrillary acidic protein (GFAP) (56) and MBP (53) were found to be significantly increased in subacute and chronic SCI, respectively. Using serological antigen selection (SAS), an unbiased cDNA phage display based technology using a human spinal cord cDNA display library, we also identified novel antibody responses directed against protein S100B, glyceraldehyde-3-phosphate dehydrogenase (GAPDH), 26S proteasome non-ATPase regulatory subunit 4 (PSMD4), adipocyte enhancer-binding protein 1 (AEBP1), and myeloma-overexpressed gene 2 (MYEOV2) in SCI samples collected at hospitalization and 3 weeks post-injury (57). A recent study that included subacute SCI patients (31 ± 1 days post-injury) identified antibodies directed against both CNS targets, including GFAP, MBP, neurofilament light (NFL), and neurofilament intermediate (NFM), and systemic antigens, such as albumin and hemoglobin (58). Interestingly, most of these autoantibodies were reported to bind modified isoforms of proteins that deviated from their expected isoelectric point or molecular weight, suggesting that the antibodies might be generated following alterations or degradation of normally exposed antigens (58).

Although autoantibodies could have protective functions, their presence following SCI has already been correlated with the development or worsening of neuropathic pain, a common adverse consequence of SCI. Recently, serum autoantibody binding in rat spinal cord tissue-based assays and primary dorsal root ganglia cell cultures was shown to be present in a subpopulation of SCI patients (with a median of 70 days post-injury) while being absent in vertebral fracture controls (12). SCI patients with autoantibody binding displayed an increased need for medication controlling neuropathic pain, which may reflect a worsening of neuropathic pain in these patients (12). Interestingly, circulating antibodies directed against GFAP and collapsing response mediator protein-2 (CRMP2) have previously also been associated with the development of neuropathic pain. The levels of anti-GFAP antibodies measured at 16 ± 7 days post-SCI were significantly increased in patients who subsequently developed neuropathic pain within 6 months post-SCI as compared to HC (56). Furthermore, the presence of autoantibodies targeting GFAP and/or CRMP2 increased the odds of developing neuropathic pain almost tenfold (56). In mouse models, the injection of SCI antibodies into the spinal cord of uninjured animals caused large necrotic inflammatory lesions and complete but transient paralysis, further suggesting their pathologic potential (59, 60). Moreover, the depletion of B cells by both a genetic knockout and anti-CD20 antibodies significantly improved locomotor activity post-injury in SCI mouse models (60, 61).

Linked with the production of autoantibodies is the overexpression of the survival receptor B-cell maturation antigen (BCMA) and the cytokines B-cell–activating factor (BAFF) and a proliferation-inducing ligand (APRIL) that was shown in PBMC of chronic SCI patients (16.5-43.7 years post-injury) compared to those of HC using microarray and real-time polymerase chain reaction (RT-PCR) analyses (62). Both APRIL and BAFF are known to bind BCMA and activate pathways involved in B cell survival, proliferation, and differentiation into memory B cells and antibody-producing plasma cells (63), which could contribute to autoreactive B cell activation. However, we did not observe increased frequencies of B cells expressing BAFF-receptor (BAFFR) or transmembrane activator calcium modulator and cyclophilin ligand interactor (TACI), two other receptors for APRIL and/or BAFF, nor increased expression levels of BAFFR or TACI on B cells from the peripheral blood of SCI patients when compared to HC (47). Furthermore, it remains unclear whether B cells and autoantibodies play a direct role in the pathophysiology of human SCI or are a byproduct of the secondary response to spinal cord damage.

The observations discussed above indicate that SCI triggers an autoimmune response in which autoreactive T and B cells and autoantibodies play a role. These cells are likely activated in response to spinal cord damage and could drive autoimmune reactions inside and outside the CNS, possibly complicating SCI recovery or causing further damage.

## 3 Suppression of the immune system

Although seemingly contradictory to the immune activation seen following SCI, systemic immunosuppression has been described in SCI patients as well (**Figure 1B**). One of the main indications of reduced immune function is the frequent occurrence of infections, predominantly affecting the respiratory and urinary tract (64–67). Respiratory tract infections are common, with a reported incidence up to 60%, during the first few days after SCI (65, 66). Contrarily, the urinary tract is the primary origin for infection during rehospitalization, affecting 34% of rehospitalized SCI patients (65, 68). Moreover, large epidemiological studies have indicated infections as the leading cause of death in the subacute and chronic phase following SCI and as independent risk factors for a worse neurological recovery (69–73). Nevertheless, underlying mechanisms explaining the high susceptibility to infections are poorly understood. In recent decades, several publications have shown changes in immune cell numbers and functions that suggest the triggering of immunosuppression following SCI.

In addition to SCI-induced inflammation, as discussed above, NK cells are frequently related to immunosuppression following SCI. One study reported decreased frequencies of CD56^bright^ NK cells and CD56^dim^ NK cells at 0-3 days post-injury compared to HC, remaining significantly suppressed up to 12 months following SCI (19). In addition, the expression of NK cell genes was significantly reduced in subacute (up to 6 months post-injury) and chronic stages (≥1 year post-injury) (19, 38). Although in another study no significant differences in total and CD56^bright^ NK cells could be demonstrated in acute SCI patients (30.3 ± 18.9 h post-injury), significantly decreased frequencies of CD56^dim^ and activated NK cells were shown compared to HC and TC (74). A statistically significant decrease in the percentage of blood NK cells following SCI has also been reported in subacute/chronic SCI patients (>3 months post-injury) compared to HC (33). In contrast, we did not find significant differences in NK cell frequencies of HC, (sub)acute (≤1 month post-injury) and subacute/chronic (>1 month post-injury) SCI patients (47). Three other studies (including samples of SCI patients >3 months, 7-120 months, and >5 years post-injury) also did not observe significant differences in NK cell numbers and frequencies in the blood and bone marrow compared to HC (31, 32, 75). Despite the inconsistent results in literature concerning the frequency of NK cells post-SCI, several research groups have confirmed a decrease in their cytotoxicity. NK cell function deficiencies after SCI were detected as early as two weeks post-injury, being ultimately suppressed at two months (76–79). These deficiencies remained present for years following SCI (33, 76–80).

SCI has also been reported to negatively affect the number and function of circulating T cells, although conflicting results have been reported concerning T cell frequencies. In one study, significant reductions in T cell numbers were evident within 24 h following SCI compared to TC (81). Cell numbers remained suppressed up to 3-4 days post-injury, but recovered significantly by the end of the first week and reached plateau control levels in later stages (105-136 days post-SCI) (81). However, since all patients were treated with a high dose of the corticosteroid methylprednisolone within 24 h after SCI, a contributing effect of this therapy cannot be excluded. In contrast, comparable frequencies of CD4^+^ and CD8^+^ T cells were demonstrated in SCI patients within 24 h of injury compared to HC (30). Moreover, we and others showed significantly increased frequencies of total T cells and CD4^+^ T cells in subacute/chronic SCI (>1 or >3 months post-injury) in comparison to (sub)acute SCI (≤1 month post-injury) and/or HC (33, 47). Two other studies reported no significant differences in circulating T cell frequencies and numbers in smaller cohorts of chronic SCI patients (7-120 months and >5 years post-injury) compared to HC (31, 32). Additionally, frequencies of total, CD4^+^, and CD8^+^ T cells in bone marrow were not significantly different between chronic SCI patients (8 months to 5 years post-injury) and HC (82). Concerning T cell function, changes have been described in multiple studies. First of all, several studies analyzed the function of lymphocytes by measuring their proliferation in response to mitogens. When comparing peripheral lymphocytes from chronic SCI patients (7-120 months post-injury) to HC, significant suppression in the lymphocyte blastogenic response to three different mitogens was reported (32). Furthermore, T cell function, as measured by lymphocyte transformation induced by a T cell mitogen, showed a significant decline starting at two weeks and reaching a minimum at three months post-injury (76–79). After that, T cell function was restored gradually over the next 3 months and stayed constant between 6 and 12 months following SCI (76–79). T cell activation, as reflected by the expression levels of IL-2 receptors, showed the same pattern in time (76–79). Lastly, the ability of T cells to kill allogeneic lymphocytes was significantly reduced in chronic SCI patients (7-40 years post-injury) compared to HC (80).

When analyzing specific T cell subsets, regulatory T cells (Tregs), that were identified as CD25^+^CD127^lo^CD4^+^ T cells that express CCR4^+^ and/or HLA-DR^+^, were significantly increased in chronic SCI (>1 year post-injury) (46). In addition, gene set enrichment analysis using an existing RNA-sequencing dataset of peripheral blood leukocytes indicated a significant increase in the expression of Tregs in acute SCI patients (30.3 ± 18.9 h post-injury) compared to HC and TC (74). However, production of IL-10, an anti-inflammatory cytokine that can be produced by Tregs, was not significantly different between chronic SCI patients (>6 months post-injury) and HC following *in vitro* stimulation of PBMC with phytohemagglutinin and lipopolysaccharide (83). Another T cell subset that has been related to immunosuppression following SCI is the CD4^+^ effector memory T cell re-expressing CD45RA (termed Temra). Significantly decreased frequencies of this cell type have been reported by us in (sub)acute SCI patients (≤1 month post-injury) compared to HC (47). Recent studies indicated Temra as specialized effector memory T cells that play a role in protective cytotoxic responses against pathogen-infected cells, indicating that decreased numbers of these cells may possibly challenge the eradication of infections (84–86).

Furthermore, alterations in the expression of chemokines and their receptors following SCI have been associated with immunosuppression. By analyzing chip-based RNA-sequencing data from peripheral blood leukocytes of acute SCI patients, expression of C-C chemokine receptor type 7 (CCR7) was found to be significantly downregulated in acute SCI patients (30.3 ± 18.9 h post-injury) compared to TC and HC, which was positively correlated with T follicular helper (Tfh) cell function (74). The authors hypothesized that following acute SCI, peripheral CCR7 is downregulated causing suppression of Tfh cells through the chemokine signaling pathway, contributing to immunosuppression (74). Moreover, a significant reduction in activated B cells was demonstrated following acute SCI (74). This reduction may also be linked to decreased CCR7 expression and impaired Tfh function, as Tfh cells play an important role in B cell maturation in the germinal center reaction. Additionally, CCR7 plays a role in effector T cell migration and positioning within secondary lymphoid organs (87). Secondly, a significant defect in the production of C-X-C motif chemokine ligand 10 (CXCL10) by monocytes in response to TLR7 and TLR9 stimulation was reported in chronic SCI patients (>6 months post-injury) with an injury level above T6 compared to HC (83). This defect in the innate immune response may contribute to the increased susceptibility to infections observed in chronic SCI.

Interestingly, it has been hypothesized that the reduced immune cell activity following SCI is attributed to a qualitative rather than a quantitative defect. In a study in which bone marrow aspirates of small cohorts of chronic SCI patients (7-40 years post-injury) and HC were analyzed, significantly reduced long-term colony formation of all hematopoietic cell lineages was reported following SCI (80). Notably, another study that also analyzed bone marrow aspirates, demonstrated that the percentage of hematopoietic stem cells was elevated in chronic SCI patients (8 months to 5 years post-injury) compared to HC, suggesting that it is the ability of the stem cells to form mature immune cells, rather than the proliferative capacity of the stem cells, that is impaired (82). The changes in the early maturation process of immune cells such as NK and T cells might in turn interfere with the development of their cytotoxic machinery and their functionality. Additionally, immune function deficits may be influenced by the level of injury, which has been demonstrated in rodent studies to be related to the anatomy of the sympathetic column of the spinal cord (88–91). In brief, upon SCI, sympathetic preganglionic neurons below the lesion level are removed from inhibitory supraspinal control, resulting in their overactivation. Consequently, the neurons release excessive amounts of norepinephrine and glucocorticoids in lymphoid organs, leading to recurrent or prolonged activation of β-2-adrenergic or glucocorticoid receptors on immune cells, promoting immunosuppressive responses (88, 91, 92). This effect is more pronounced for lesions at cervical or high thoracic spinal segments since the majority of sympathetic innervation of immunologically relevant organs originates from preganglionic neurons situated below the fifth thoracic segment (T5), both for experimental animal models and for humans (89). Indications for this phenomenon were also obtained in human studies. In motor complete SCI patients, injuries at high (T1–4) relative to mid (T4-8) thoracic levels and at mid relative to low (T9-12) thoracic levels were independently associated with an increased risk for pneumonia following SCI (89). In another study, the mean killing capacity of NK cells was significantly reduced in subacute/chronic tetraplegic SCI patients, whereas no significant difference was observed in subacute/chronic paraplegic (at or below T10) SCI patients (>3 months post-injury) compared to HC (75). Although no statistically significant difference could be demonstrated for neutrophils, the phagocytic activity of neutrophils showed a decreasing trend in the tetraplegic group, whereas their activity was unchanged in paraplegic patients when compared with HC (75). In contrast, other studies did not observe differences in neutrophil oxygen consumption, eosinophil activation, and the cytotoxic capability of NK and T cells between HC, chronic tetraplegics (cervical segment (C)5-7), and chronic paraplegics (T5-10) (>5 years and 7-40 years post-injury) (31, 80). In addition, no significant difference in NK cytotoxicity was observed between subacute/chronic SCI patients with injuries at T6 and above and those with injuries below T6 (>3 months post-injury) (33).

Altogether, the studies discussed above hypothesize that changes in lymphocyte frequencies and functions contribute to the increased risk of infections following SCI. These changes are thought to result from impaired maturation of immune cells and have been related to lesions at higher levels along the spinal column, potentially affecting immune regulation and response.

## 4 Conclusion

SCI adversely affects the immune system, contributing to changes in immune system composition and function both at the site of injury and in the periphery. Seemingly contrasting findings of peripheral immune system activation and suppression have been reported in traumatic SCI patients and these phenomena seem to emerge simultaneously in both (sub)acute and chronic stages following the injury. In response to SCI, a systemic inflammatory host response is triggered. In the (sub)acute stages, immune activation manifests prominently through increased neutrophil and monocyte frequencies and their enhanced oxidative and migratory capabilities, whereas in chronic stages, increased levels of cytokines, acute phase proteins, TLR signaling genes, and T and B cell subsets related to activation and inflammation were reported. In parallel, SCI-induced immune suppression develops quickly and extends into chronic stages, possibly to avert autoimmunity against self-antigens that are released or expressed following SCI. Immune suppression post-SCI is most importantly marked by significant decreases in T cell and NK cell frequencies and functions and is influenced by the level of injury, although this should be confirmed in further studies. Notably, a similar paradox of immune activation and suppression has been described in other conditions involving neuronal injury, including stroke and traumatic brain injury (93–95). Additionally, deficient or ineffective initiation of SCI-induced immune suppression may result in autoimmunity, in which MBP-reactive and memory T cells, B cells, and autoantibodies have been reported to play a role. Presumably, a balance between immune system activation and suppression is necessary to reestablish homeostasis in the affected tissues. As a consequence, an imbalance may result in an exaggerated manifestation of one of both responses. The resulting peripheral changes in immune function could complicate SCI recovery or lead to an aberrant immune reaction long after the initial trauma, for example, leading to an increased susceptibility to infections or systemic organ failure.

The peripheral changes after SCI have only been studied in small patient cohorts and numerous clinical characteristics, such as the spinal injury level, the time after injury, and the injury severity, can influence the progression of SCI and study results. Therefore, future studies should focus on large patient cohorts with varied clinical characteristics that accurately represent the diversity of the SCI patient population. Furthermore, the association of clinical characteristics with peripheral immune reactions following SCI should be investigated. A more detailed understanding of alterations in immune cell function, their temporal occurrence, and potential correlations with clinical characteristics could facilitate the clinical assessment and therapeutic management of SCI. Additionally, many of the reviewed publications lack information on the included treatments that could potentially affect the immune system. In this regard, whilst their clinical utility remains controversial, traumatic SCI patients are frequently treated with immunomodulatory drugs, including corticosteroids such as methylprednisolone (7, 96). Other therapeutic options for SCI that interfere with the immune response include non-steroidal anti-inflammatory drugs (NSAIDs) and antibiotics (7). In case these immunotherapies are not listed as exclusion criteria, their potential impact on study outcomes cannot be excluded. Hence, participants receiving treatments that impact the immune response should either be excluded or their treatments should be clearly mentioned in future studies. Moreover, examining the correlation between these treatments and the immune response to SCI would be valuable.

Finally, the reports discussed in this review highlight the need for the development of novel, more efficient therapeutic approaches to address the immune system-related consequences of SCI that extend beyond the spinal cord. Notably, it is important that these strategies control peripheral immunosuppression without aggravating immune cell-mediated damage to the spinal cord and other organs. Moreover, therapies that could ameliorate SCI recovery and prevent further damage by addressing autoreactivity merit attention. Reevaluating peripheral changes in immune system composition and function following SCI from these perspectives could open doors to more specific and personalized therapies for SCI.
