# Supplementary material for: Peripheral immune reactions following human traumatic spinal cord injury: the interplay of immune activation and suppression
Source: Front Immunol. 2024 Nov 27;15:1495801. doi: 10.3389/fimmu.2024.1495801 (PMC11631733; doi:10.3389/fimmu.2024.1495801)
Supplement: Supplementary file 1 [file Table1.docx]

Supplementary Material

**Supplementary Table 1:** Alterations in cellular components of the peripheral immune system following SCI.

| *Technique* | *Alterations* | *Patient characteristics* | *Inclusion of immunotherapy-treated patients* | *Ref.* |
| --- | --- | --- | --- | --- |
| **Hematopoietic progenitor cells** | | | | |
| Flow cytometry | Frequencies ↑ in bone marrow | cSCI (8 m to 5 ypi); n=16 vs. n=10 HC | N.a. | (82) |
| Bone marrow progenitor cell assay | Ability of progenitor cells to form hematopoietic cell lineages, including dendritic cells ↓ in bone marrow | cSCI (7-40 ypi); tp and pp vs. HC, n=6 each | No | (80) |
| **All blood cells** | | | | |
| RNA sequencing | Expression of HMGB1 ↑ at 0-3 dpi until 6 mpi; expression of TLR2 and -4 ↑ at 0-3 dpi and 6 mpi | a/s/cSCI (0-3 d to 12 mpi); n=10 vs. n=9 HC | N.a. | (19) |
|  | Expression of TLR signaling pathways and HMGB1 ↑ | cSCI (≥1 ypi); n=31 vs. n=26 HC | N.a. | (38) |
| **Leukocytes** | | | | |
| WBC differential count | Numbers elevated vs. reference values in blood | aSCI (3.5±1 hpi); n=9 and n=6 TC^a^ | No | (16) |
|  | Numbers ↑ in blood | a/sSCI (<1 wpi); n=21 vs. n=11 TC^b^ | Yes | (17) |
|  | Numbers not significantly different in blood | cSCI (>5 ypi); tp and pp vs. HC, n=6 each | N.a. | (31) |
| Flow cytometry |  | s/cSCI (>3 mpi); n=36 vs. n=34 HC | No | (33) |
|  |  | cSCI (7-120 mpi); n=5 vs. n=5 HC | No | (32) |
| RNA sequencing | CCR7 expression ↓ in blood | a/sSCI (5-97 hpi); n=38 vs. n=10 TC^c^ and n=10 HC | N.a. | (74) |
| DCFH-DA assay | Free radical formation ↑ vs. TC^a^ at 12 h, 24 h, 1 w, and 2 wpi and vs. HC at 6 h to 2 wpi in blood | a/sSCI (6 h to 2 wpi); n=9 vs. n=6 TC^a^ and n=10 HC | No | (16) |
| Myeloperoxi-dase assay | Myeloperoxidase activity ↑ vs. TC^a^ at 24 h to 1 wpi and vs. HC at 6 h to 2 wpi in blood |  |  |  |
| Western blot assay | Expression of gp91^phox^ and iNOS ↑ vs. TC^a^ and HC in blood | aSCI (24 hpi); n=4 vs. n=4 TC^a^ and n=4 HC |  |  |
| **Granulocytes** | | | | |
| Flow cytometry | Numbers not significantly different in blood | a/sSCI (<24 h to 136 dpi); n=16 vs. n=10 TC^d^ | Yes | (81) |
|  |  | s/cSCI (>3 mpi); n=10 tp vs. n=10 HC and n=8 pp vs. n=8 HC | No | (75) |
|  |  | s/cSCI (>3 mpi); n=36 vs. n=34 HC | No | (33) |
|  | Frequencies not significantly different in blood | cSCI (7-120 mpi); n=5 vs. n=5 HC | No | (32) |
|  | Frequencies not significantly different in bone marrow | cSCI (>5 ypi); tp and pp vs. HC, n=6 each | N.a. | (31) |
|  | Levels of cellular adhesion molecules ↓ (α3, α4, CD11a, CD18, and CD8) in blood | SCI (n.a.); n=31 vs. n=n.a. HC | N.a. | (76) |
| Neutrophils | | | | |
| WBC differential count | Numbers ↑ vs. clinical reference range at admission and/or 1 dpi in exploration and validation cohort and ↓ vs. admission and 1 dpi at 3 and 7 dpi in exploration cohort in blood | a/sSCI (0-8 dpi); n=163 in exploration cohort and n=49 in validation cohort | Yes | (18) |
|  | Numbers elevated vs. reference values in blood | aSCI (3.5±1 hpi); n=9 and n=6 TC^a^ | No | (16) |
|  | Numbers not significantly different in blood | cSCI (>5 ypi); tp and pp vs. HC, n=6 each | N.a. | (31) |
| Flow cytometry | Surface expression of CD62L ↓ vs. HC at 6 h to 1 wpi; surface expression of α4 ↑ vs. TC^a^ at 12 h to 1 wpi and vs. HC at 12-24 h and 1 wpi; surface expression of CD11d ↑ vs. TC^a^ at 12-48 h and 2 wpi and vs. HC at 48 h and 2 wpi; surface expression of CD11b ↑ vs. TC^a^ and HC at 1 wpi in blood | a/sSCI (6 h to 2 wpi); n=9 vs. n=6 TC^a^ and n=10 HC | No | (27) |
| Dihydrorhoda-mine 123 staining | Oxidative activity ↑ vs. TC^a^ at 12-24 h and 1 wpi and vs. HC at 12-48 h and 1 wpi in blood | a/sSCI (6 h to 2 wpi); n=9 vs. n=6 TC^a^ and n=10 HC | No | (16) |
| Neutrophil functional assay | Functional ability to phagocytize bacteria not significantly different for tp and pp in blood | s/cSCI (>3 mpi); n=10 tp vs. n=10 HC and n=8 pp vs. n=8 HC | No | (75) |
|  | Migration and oxygen consumption not significantly different in blood | cSCI (>5 ypi); tp and pp vs. HC, n=6 each | N.a. | (31) |
| Eosinophils | | | | |
| Flow cytometry | Numbers not significantly different in blood | s/cSCI (>3 mpi); n=36 vs. n=34 HC | No | (33) |
| Radioimmuno-assay and flow cytometry | Activation not significantly different in blood | cSCI (>5 ypi); tp and pp vs. HC, n=6 each | N.a. | (31) |
| Basophils | | | | |
| Flow cytometry | Numbers not significantly different in blood | s/cSCI (>3 mpi); n=36 vs. n=34 HC | No | (33) |
| **Total PBMC** | | | | |
| Microarray analysis | Expression of BCMA, APRIL, and BAFF ↑ in blood | cSCI (16.5-43.7 ypi); n=6 vs. n=5 HC | N.a. | (62) |
| qPCR |  | cSCI (1.1-36.5 ypi); n=10 vs. n=6 HC |  |  |
| ELISA | Production of IL-10 in response to PHA and LPS not significantly different in blood | cSCI (>6 mpi); n=17 vs. n=13 HC | No | (83) |
| **Monocytes** | | | | |
| WBC differential count | Numbers not elevated vs. reference values in blood | aSCI (3.5±1 hpi); n=9 and n=6 TC^a^ | No | (16) |
|  | Numbers ↑ vs. admission at 1 and 7 dpi in exploration cohort; numbers not significantly different in validation cohort in blood | a/sSCI (0-8 dpi); n=163 exploration cohort and n=49 validation cohort | Yes | (18) |
|  | Numbers not significantly different in blood | cSCI (>5 ypi); tp and pp vs. HC, n=6 each | N.a. | (31) |
| Flow cytometry | Surface expression of CD62L ↓ vs. HC at 6 h to 2 wpi; surface expression of α4 ↑ vs. TC^a^ at 24 h to 1 wpi; surface expression of CD11d ↑ vs. TC^a^ at 12-48 h and 2 wpi and vs. HC at 12 h, 48 h, and 2 wpi; surface expression of CD11b ↑ vs. TC^a^ at 24-48 h and 1 wpi and vs. HC at 12-48 h and 1-2 wpi in blood | a/sSCI (6 h to 2 wpi); n=9 vs. n=6 TC^a^ and n=10 HC | No | (27) |
|  | Numbers not significantly different vs. TC^d^; numbers ↑ vs. 24 h at 6-8 d and 25-30 dpi and vs. 3-4 d at 6-8 dpi in blood | a/sSCI (<24 h to 136 dpi); n=16 vs. n=10 TC^d^ | Yes | (81) |
|  | Frequencies of classical (CD14^+^CD16^-^) and intermediate (CD14^+^CD16^+^) monocytes ↑ at 0-3 dpi; frequencies of non-classical (CD14^-/lo^CD16^+^) monocytes not significantly different in blood | a/s/cSCI (0-3 d to 12 mpi); n=7 vs. n=12 HC | N.a. | (19) |
|  | Frequencies not significantly different in blood | a/sSCI (≤1 mpi) and s/cSCI (>1 mpi); n=18 vs. n=18 HC | No | (47) |
|  | Numbers not significantly different in blood | s/cSCI (>3 mpi); n=36 vs. n=34 HC | No | (33) |
|  |  | s/cSCI (>3 mpi); n=10 tp vs. n=10 HC and n=8 pp vs. n=8 HC | No | (75) |
|  | Frequencies of CXCL10^+^ monocytes ↓ following TLR7 and TLR9 stimulation | cSCI (>6 mpi); n=7 vs. n=7 HC | No | (83) |
|  | Frequencies not significantly different in blood | cSCI (7-120 mpi); n=5 vs. n=5 HC | No | (32) |
| RNA sequencing | Expression of genes associated with monocyte activation ↑ at 0-3 dpi and remained elevated during 6 mpi in blood | a/s/cSCI (0-3 d to 12 mpi); n=10 vs. n=9 HC | N.a. | (19) |
| Dihydrorhoda-mine 123 staining | Oxidative activity ↑ vs. TC^a^ at 12-24 h and 1 wpi and vs. HC at 12-48 h and 1 wpi in blood | a/sSCI (6 h to 2 wpi); n=9 vs. n=6 TC^a^ and n=10 HC | No | (16) |
| **Lymphocytes** | | | | |
| WBC differential count | Numbers ↓ vs. admission at 1 and 3 dpi and resolved by 7 dpi in exploration cohort; numbers ↓ vs. clinical reference range at 0 and 3 dpi in validation cohort in blood | a/s SCI (0-8 dpi); n=163 in exploration cohort and n=49 in validation cohort | Yes | (18) |
|  | Numbers ↓ in blood | sSCI (1 wpi); n=21 vs. n=11 TC^b^ | Yes | (17) |
| Flow cytometry | Numbers not significantly different in blood | s/cSCI (>3 mpi); n=36 vs. n=34 HC | No | (33) |
|  |  | s/cSCI (>3 mpi); n=10 tp vs. n=10 HC and n=8 pp vs. n=8 HC | No | (75) |
|  | Frequencies not significantly different in blood | cSCI (7-120 mpi); n=5 vs. n=5 HC | No | (32) |
|  | Numbers not significantly different in blood and frequencies not significantly different in bone marrow | cSCI (>5 ypi); tp and pp vs. HC, n=6 each | N.a. | (31) |
| Lymphocyte functional assay | Proliferation in response to concanavalin A, phytohemagglutinin, and pokeweed mitogen ↓ in blood | cSCI (7-120 mpi); n=5 vs. n=5 HC | No | (32) |
| T cells | | | | |
| Flow cytometry | Frequencies of CD4^+^ and CD8^+^ T cells not significantly different in blood | aSCI (≤24 hpi); n=21 vs. n=20 HC | No | (30) |
|  | Numbers ↓ vs. TC^d^ at 24 hpi and 3-4 dpi; numbers ↑ vs. 24 h at 6-136 dpi; numbers ↑ vs. 3-4 d at 6-30 dpi in blood | a/sSCI (<24 h to 136 dpi); n=16 vs. n=10 TC^d^ | Yes | (81) |
|  | Frequencies of CD4^+^ T cells ↓ at 3 mpi; frequencies of CD8^+^ T cells not significantly different; frequencies of activated (HLA-DR^+^) CD4^+^ and CD8^+^ T cells ↑ at 3, 6, and 12 mpi in blood | a/s/cSCI (0-3 d to 12 mpi); n=7 vs. n=12 HC | N.a. | (19) |
|  | Frequencies of total T cells and CD4^+^ T cells ↑ in s/cSCI vs. a/sSCI and HC; frequencies of CD8^+^ T cells not significantly different; frequencies of CD4^+^ CD45RA^+^ CCR7^-^ T cells ↓ in a/sSCI vs. HC in blood | a/sSCI (≤1 mpi) and s/cSCI (>1 mpi); n=18 vs. n=18 HC | No | (47) |
|  | Frequencies of total T cells and CD4^+^ T cells ↑; frequencies of CD8^+^ T cells not significantly different in blood | s/cSCI (>3 mpi); n=36 vs. n=34 HC | No | (33) |
|  | Numbers not significantly different in blood | s/cSCI (>3 mpi); n=10 tp vs. n=10 HC and n=8 pp vs. n=8 HC | No | (75) |
|  | Frequencies not significantly different in blood | cSCI (7-120 mpi); n=5 vs. n=5 HC | No | (32) |
|  | Frequencies of total T cells and CD4^+^ and CD8^+^ T cells not significantly different in bone marrow | cSCI (8 m to 5 ypi); n=16 vs. n=7 HC | N.a. | (82) |
|  | Frequencies of total T cells and CD4^+^ T cells ↓; frequencies of CD8^+^ T cells not significantly different; frequencies of HLA-DR^+^ CD4^+^ T cells ↑; frequencies of CD25^+^ CD127^lo^ CD4^+^ T cells that express CCR4^+^ and/or HLA-DR^+^ ↑ in blood | cSCI (>1 ypi); n=19-22 vs. n=11 HC | N.a. | (46) |
|  | Concentrations not significantly different in bone marrow; numbers not significantly different in blood | cSCI (>5 ypi); tp and pp vs. HC, n=6 each | N.a. | (31) |
| RNA sequencing | Expression of activated CD8^+^ ↓; expression of regulatory T cells ↑; expression of activated CD4^+^ not significantly different vs. TC^c^+HC in blood | a/sSCI (5-97 hpi); n=38 vs. n=10 TC^c^ and n=10 HC | N.a. | (74) |
|  | Abundance of CD8^+^ T cells ↓ vs. TC^c^ and HC in blood |  |  |  |
|  | Expression of genes linked to development, proliferation, and survival of T cells ↓ at 0-3 dpi and remained reduced during 6 mpi; expression of genes linked to T cell activation ↑ at 0-3 dpi in blood | a/s/cSCI (0-3 d to 12 mpi); n=10 vs. n=9 HC | N.a. | (19) |
| IL-2R assay | Activation as reflected by IL-2R levels ↓ between 2 wpi and 3 mpi and ↑ between 3 and 6 mpi in blood | s/cSCI (0.5-12 mpi); n=34 vs. n=n.a. HC, n=34 vs. n=24-32 HC, n=54 vs. n=32 HC, n=49 vs. 32 HC | N.a. | (76-79) |
| Lymphocyte functional assay | Proliferation in response to leucoagglutinin (T cell mitogen) ↓ between 2 wpi and 3 mpi and ↑ between 3 and 6 mpi in blood |  |  |  |
|  | Cytotoxic capability ↓ in blood | cSCI (7-40 ypi); n=6 tp and n=6 pp vs. n=6 HC | No | (80) |
| Cell culture-based assay | Frequency of MBP-reactive T cells not significantly different in blood | cSCI (>5 ypi); n=15 vs. n=14 MS and n=11 HC | No | (52) |
|  | Proliferation in response to MBP ↑ in blood | cSCI (>10 ypi); n=12 vs. n=18 HC | N.a. | (53) |
| B cells | | | | |
| Flow cytometry | Frequencies not significantly different in blood | aSCI (≤24 hpi); n=21 vs. n=20 HC | No | (30) |
|  | Numbers ↓ vs. TC^d^ at 24 hpi; numbers ↑ vs. 3-4 d at 6-8 dpi in blood | a/sSCI (<24 h to 136 dpi); n=16 vs. n=10 TC^d^ | Yes | (81) |
|  | Frequencies of B cells and major subtypes (CSM, NCSM, naive, transitional, and DN B cells and plasmablasts) not significantly different in blood | a/s/cSCI (0-3 d to 12 mpi); n=7 vs. n=12 HC | N.a. | (19) |
|  | Frequencies of total B cells not significantly different; frequencies of naive B cells ↓ in s/cSCI vs. a/sSCI; frequencies of CSM B cells ↑ in s/cSCI vs. a/sSCI; frequencies of IgM^+^ B cells ↓ in s/cSCI vs. a/sSCI; frequencies of IgA^+^ B cells ↑ in s/cSCI vs. a/sSCI and HC; frequencies of CD74^+^ B cells ↑ in s/cSCI and a/sSCI vs. HC in blood | a/sSCI (≤1 mpi) and s/cSCI (>1 mpi); n=18 vs. n=18 HC | No | (47) |
|  | Numbers not significantly different in blood | s/cSCI (>3 mpi); n=10 tp vs. n=10 HC and n=8 pp vs. n=8 HC | No | (75) |
|  | Frequencies not significantly different in blood | s/cSCI (>3 mpi); n=36 vs. n=34 HC | No | (33) |
|  |  | cSCI (7-120 mpi); n=5 vs. n=5 HC | No | (32) |
|  | Frequencies not significantly different in bone marrow | cSCI (8 m to 5 ypi); n=16 vs. n=7 HC | N.a. | (82) |
|  | Numbers of B cells not significantly different in blood; concentrations of B cells not significantly different in bone marrow; frequencies of plasma cells not significantly different in bone marrow | cSCI (>5 ypi); tp and pp vs. HC, n=6 each | N.a. | (31) |
| RNA sequencing | Expression of activated, immature, and memory B cells ↓ vs. TC^c^+HC in blood | a/sSCI (5-97 hpi); n=38 vs. n=10 TC^c^ and n=10 HC | N.a. | (74) |
|  | Abundance of naive B cells ↓ vs. TC^c^ and HC in blood |  |  |  |
| Radial immune-diffusion | Function as reflected by IgG levels ↓ in blood | cSCI (7-40 ypi); tp and pp vs. HC, n=6 each | No | (80) |
| NK cells | | | | |
| Flow cytometry | Frequencies of total NK cells ↑; frequencies of CD56^bright^ NK cells ↓; frequencies of activated NK cells ↑ in blood | aSCI (≤24 hpi); n=21 vs. n=20 HC | No | (30) |
|  | Frequencies of CD56^bright^ and CD56^dim^ NK cells ↓ at 0-3 dpi and remained reduced during 12 mpi in blood | a/s/cSCI (0-3 d to 12 mpi); n=7 vs. n=12 HC | N.a. | (19) |
|  | Frequencies not significantly different in blood | a/sSCI (≤1 mpi) and s/cSCI (>1 mpi); n=18 vs. n=18 HC | No | (47) |
|  | Frequencies ↓ vs. HC in blood | s/cSCI (>3 mpi); n=36 vs. n=34 HC | No | (33) |
|  | Numbers not significantly different in blood | s/cSCI (>3 mpi); n=10 tp vs. n=10 HC and n=8 pp vs. n=8 HC | No | (75) |
|  | Frequencies not significantly different in blood | cSCI (7-120 mpi); n=5 vs. n=5 HC | No | (32) |
|  | Frequencies not significantly different in bone marrow | cSCI (8 m to 5 ypi); n=16 vs. n=7 HC | N.a. | (82) |
|  | Numbers not significantly different in blood; concentrations not significantly different in bone marrow | cSCI (>5 ypi); tp and pp vs. HC, n=6 each | N.a. | (31) |
| RNA sequencing | Expression of CD56^dim^ NK cells ↓; expression of total NK cells and CD56^bright^ NK cells not significantly different vs. TC^c^+HC in blood | a/sSCI (5-97 hpi); n=38 vs. n=10 TC^c^ and n=10 HC | N.a. | (74) |
|  | Abundance of activated NK cells ↓ vs. TC^c^ and HC in blood |  |  |  |
|  | Expression of canonical and signal transduction NK cell genes ↓ at 0-3 dpi and remained reduced during 6 mpi in blood | a/s/cSCI (0-3 d to 12 mpi); n=10 vs. n=9 HC | N.a. | (19) |
|  | Expression not significantly different; expression of cytotoxic NK cell genes ↓ in blood | cSCI (≥1 ypi); n=31 vs. n=26 HC | N.a. | (38) |
| NK cell cytotoxicity assay | Cytotoxic capability ↓ by 2 mpi and between 7 and 9 mpi; mean value for cytotoxic capability ↓ in blood | s/cSCI (0.5-12 mpi); n=49 vs. n=n.a. HC, n=34 vs. n=24-32 HC, n=54 vs. n=32 HC, n=49 vs. n=32 HC | N.a. | (76-79) |
|  | Cytotoxic capability ↓ vs. HC; cytotoxic capability not significantly different in T6 and above vs. below T6 in blood | s/cSCI (>3 mpi); n=36 vs. n=34 HC; n=26 injury at T6 and above vs. n=10 injury below T6 | No | (33) |
|  | Cytotoxic capability ↓ for tp and not significantly different for pp in blood | s/cSCI (>3 mpi); n=10 tp vs. n=10 HC and n=8 pp vs. n=8 HC | No | (75) |
|  | Cytotoxic capability not significantly different in blood | cSCI (7-120 mpi); n=5 vs. n=5 HC | No | (32) |
|  | Cytotoxic capability ↓ in blood | cSCI (7-40 ypi); tp and pp vs. HC, n=6 each | No | (80) |

^a^ Trauma controls with vertebrae or long bone fractures without CNS injury; ^b^ Trauma controls with isolated spine trauma without CNS injury; ^c^ Trauma controls with undefined trauma without CNS injury; ^d^ Trauma controls with (lower limb) surgery without CNS injury. Abbreviations: *APRIL, a proliferation-inducing ligand; aSCI, acute spinal cord injury (≤24 hours post-injury); BAFF,* *B-cell activating factor; BCMA, B-cell maturation antigen; CCR, C-C chemokine receptor type 7; cSCI, chronic spinal cord injury (≥6 months post-injury); CSM, class-switched memory; CXCL10, C-X-C motif chemokine ligand 10; DCFH-DA, 2′-7′-dichlorofluorescein-diacetate; DN, double negative; dpi, days post-injury; ELISA, Enzyme-Linked Immunosorbent Assay; HC, healthy controls;* *HLA-DR,* *Human Leukocyte Antigen DR isotype; HMGB1, High Mobility Group Box 1 protein;* *hpi, hours post-injury; IL-2R, interleukin-2 receptor; iNOS, inducible nitric oxide synthetase; LPS, lipopolysaccharide; MBP, myelin basic protein; mpi, months post-injury; MS, multiple sclerosis**; n, sample size; n.a., not available; NCSM, non-class switched memory; NK, natural killer; PBMC, peripheral blood mononuclear cells; PHA, phytohemagglutinin; pp, paraplegics; qPCR, quantitative polymerase chain reaction; sSCI, subacute spinal cord injury; TLR, Toll-like receptor; tp, tetraplegics; T6, thoracic segment six; WBC, white blood cell; wpi, weeks post-injury; ypi, years post-injury; ↑, increased; ↓, decreased.*
