# Supplementary material for: Peripheral immune reactions following human traumatic spinal cord injury: the interplay of immune activation and suppression
Source: Front Immunol. 2024 Nov 27;15:1495801. doi: 10.3389/fimmu.2024.1495801 (PMC11631733; doi:10.3389/fimmu.2024.1495801)
Supplement: Supplementary file 2 [file Table2.docx]

Supplementary Material

**Supplementary Table 2:** Alterations in non-cellular components of the peripheral immune system following SCI.

| *Technique* | *Alterations* | *Patient characteristics* | *Inclusion of immunotherapy-treated patients* | *Ref.* |
| --- | --- | --- | --- | --- |
| **Serum** | | | | |
| ELISA | IL-6, TNF-α, and anti-GM1 (IgG) levels ↑ | s/cSCI (2 to >52 wpi); n=56 vs. n=35 HC | No | (34) |
|  | TNF-α and anti-GM1 (IgM) levels ↑; anti-GM1 (IgG) levels not significantly different | cSCI (>12 mpi); n=24 vs. n=26 HC | N.a. | (35) |
|  | CRP levels ↑ | cSCI (1.2-27.7 ypi); n=69 vs. n=29 HC | No | (42) |
|  | IL-6 and CRP levels elevated vs. reference values | cSCI (>3 ypi); n=22 | Yes | (44) |
|  | CRP levels ↑; IL-6 and TNF-α levels not significantly different | cSCI (mean 10.97±8.18 ypi); n=34 vs. n=10 HC | N.a. | (37) |
|  | anti-MBP (IgG) levels ↑ | cSCI (>10 ypi); n=12 vs. n=18 HC | N.a. | (53) |
| 2D-Western blot and mass spectrometry | IgG and IgM autoantibodies ↑ against sixteen targets encoded by known proteins: PPIA, PGK1, PEBP1, NFL, MBP, HSP7C, HBB, HBA, GFAP, G3P, ENOA, CAH2, DHE3, ATPA, ALBU, and NFM | sSCI (median time of 31±1 dpi); n=52 vs. n=16 HC | No | (58) |
| Radial immune-diffusion | CRP levels ↑ in infected vs. noninfected and in noninfected vs. HC | s/cSCI (>3 mpi); n=19 infected and n=34 noninfected vs. n=10 HC | No | (43) |
| Hematology measures | ESR levels ↑ in infected vs. noninfected and in noninfected vs. HC |  |  |  |
| Protein electrophoresis | A1G levels ↑ in infected vs. noninfected and in noninfected vs. HC |  |  |  |
| **Plasma** | | | | |
| ELISA | MIF levels ↑ | aSCI (median 16 hpi); n=207 vs. n=100 HC | N.a. | (48) |
|  | HMGB1 levels ↑ in a/sSCI and cSCI vs. HC | a/sSCI (≤1 wpi) and cSCI (≥1 ypi); n=16 a/sSCI and n=47 cSCI vs. n=51 HC | N.a. | (40) |
|  | IL-6 levels not significantly different | cSCI (1-46 ypi); n=70 vs. n=20 HC | N.a. | (36) |
| Multiplex assay | MIF levels ↑ at 0-3, 4-7, and 8-11 dpi | a/sSCI (0-15 dpi); n=18 vs. n=18 HC | Yes | (49) |
|  | MIF levels ↑ | cSCI (≥1 ypi); n=22 vs. n=19 HC | Yes | (50) |
| Serological antigen selection | IgG autoantibodies ↑ against 5 antigenic targets encoded by known proteins: S100B, GAPDH, PSMD4, AEBP1, and MYEOV2 | a/sSCI (at hospitalization or 3 wpi); n=10 vs. n=9 HC | N.a. | (57) |
| Capillary Westerns | anti-GFAP (IgG) levels ↑ | sSCI (16±7 dpi); n=38 vs. n=19 HC | N.a. | (56) |

Abbreviations: *AEBP1, adipocyte enhancer-binding protein 1; ALBU, albumin; aSCI, acute spinal cord injury (≤24 hours post-injury); ATPA, ATP synthase subunit alpha mitochondrial; A1G, alpha-1 globulin; CAH2, carbonic anhydrase 2; CRP, C-reactive protein; cSCI, chronic spinal cord injury (≥6 months post-injury); DHE3, glutamate dehydrogenase 1;* *dpi, days post-injury; ELISA, Enzyme-Linked Immunosorbent Assay; ENOA, alpha-enolase; ESR, erythrocyte sedimentation rate; GADPH, glyceraldehyde-3-phosphate dehydrogenase; GFAP, glial fibrillar acidic protein; GM1, monosialotetrahexosylganglioside; G3P, glyceraldehyde-3-phosphate dehydrogenase; HBA, hemoglobin subunit alpha; HBB, hemoglobin subunit beta; HC, healthy controls; HMGB1, High Mobility Group Box 1 protein; hpi, hours post-injury; HSP7C, heat shock cognate 71 KDa protein; Ig, immunoglobulin; IL-6, interleukin 6; MBP, myelin basic protein;* *mpi, months post-injury; MYEOV2, myeloma-overexpressed gene 2; n, sample size; n.a., not available; NFL, neurofilament light; NFM, neurofilament intermediate; PEBP1, phosphatidylethanolamine binding protein 1; PGK1, phosphoglycerate kinase 1; PPIA, peptidylprolyl isomerase 1; PSMD4, 26S proteasome non-ATPase regulatory subunit 4;* *sSCI, subacute spinal cord injury; S100B, protein S100-B; TLR, Toll-like receptor; TNF-α, tumor necrosis factor-α; 2D, two-dimensional;* *wpi, weeks post-injury; ↑, increased; ↓, decreased.*
